# A Case of Emphysema

**Published:** 1845-01

**Authors:** E. W. Harris

**Affiliations:** Cape Girardeau county, Mo.


Art. II.—*A Case of Emphysema.* By E. W. Harris, M. D., of Cape Girardeau county, Mo.

The report of Dr. Girdner's case of Emphysema induces me to publish the particulars of one similar to it in every respect.

Mr. C——, aged 40 years, received a gun-shot wound in 1841, the ball passing through the upper arm, and fracturing a rib, the lung being wounded by a spicula of bone.

I was called 24 hours after the accident occurred, and found him under the care and management of a German, who called himself *doctor* (one of the cold-water tribe). I found the patient enveloped in sheets—sheets, bed and all, deluged with cold-water; almost pulseless; the whole surface cold, and of a leaden hue; every part of the body enormously distended, the scrotum being swollen to the ordinary size of the adult head; every portion of cellular membrane seemed to be filled with air, the crepitus of which was distinct and audible.

The wet clothing was speedily removed, dry warmth applied, and diffusible stimulants given; under which, reaction was established in about six hours.

My German friend said to me, that "it ish de dhropsies; you must tap him." To the latter I assented, and punctured the scrotum, and the skin in some half dozen other places, from which punctures the air could be heard to escape with considerable force. The German, offended that it was not *water*, left. The patient, much relieved, set out in two days for his home, in Illinois, and in a short time recovered perfectly.

An old hunter, who saw the above case, enquired of me the cause of the swelling, stating that he had frequently seen the same in deer when shot in the 'lights.'

November, 1844.

